# Hybrid warfare and counter-terrorism medicine

**DOI:** 10.1007/s00068-023-02230-y

**Published:** 2023-02-10

**Authors:** Derrick Tin, Dennis G. Barten, Fredrik Granholm, Pavlo Kovtonyuk, Frederick M. Burkle, Gregory R. Ciottone

**Affiliations:** 1 Department of Emergency Medicine, Beth Israel Deaconess Medical Center and Harvard Medical School, Boston, USA; 2 Department of Emergency Medicine, VieCuri Medical Center, Venlo, The Netherlands; 3 Department of Emergency Medicine and EMS, Sundsvall County Hospital, Sundsvall, Sweden; 4 Ukrainian Healthcare Center, Kiev, Ukraine; 5 Harvard Humanitarian Initiative, Harvard University, Cambridge, USA; 6 Department of Critical Care Medicine, University of Melbourne, Melbourne, Australia

**Keywords:** Disaster medicine, Counter Terrorism Medicine, Hybrid warfare, Ukraine, Pre hospital medicine

## Abstract

**Introduction:**

March 9, 2022. An airstrike by Russian forces destroying a maternity hospital in Mariupol, Ukraine. The image of a severely injured pregnant woman covered in blood being stretchered away against the backdrop of destroyed buildings. Mutterings of the use of chemical weapons. This paper is a primer for healthcare personnel and health systems on hybrid warfare and counter-terrorism medicine.

**Discussion:**

While recent events and images arising from conflicts around the world represent a cruel hallmark in today’s history, attacks against healthcare facilities and innocent civilians are not new and continue to be perpetrated around the world. In war, the Geneva Convention protects civilians and healthcare institutions from harm but when war crimes are being committed and civilians knowingly targeted, parallels from a healthcare perspective can be drawn with terrorism events. Increasingly, civilian institutions and in particular the healthcare sector, are drawn into such conflicts and understanding the health system impact of hybrid warfare and other asymmetrical attack methods is of great importance.

**Conclusion:**

The field of Counter-Terrorism Medicine (CTM) explores the healthcare impacts of intentional, man-made attacks and much recent research and discussions around this topic are extremely relevant and applicable not just to the ongoing hybrid war in Ukraine, but to today’s threat climate all around us.

## Introduction to counter-terrorism medicine

Since the “War on Terror” began in 2001, much political and security focus has been around transnational terrorism. 2014 saw a peak in global terrorist attacks, with over 16,000 documented events that year alone [1]. While the healthcare impacts of terrorist attacks can be profound for the individual victims and their communities, this topic remains an under researched and under discussed sub-branch of disaster medicine. Health systems remain unprepared to deal with intentional mass casualty events, research remains lacking in trying to understand the extent of psychosocial and economic impact of attacks, and civilian sector medics are ill equipped with the competencies required to respond to and manage victims of terrorist attacks [2].

War and conflict today, particularly in the urban setting, blur the lines between traditional, guerrilla and hybrid warfare and terrorism. Because untrained civilians and insurgency groups taking up arms may not understand or respect the rules of engagement, civilian medical providers may find themselves in unfamiliar and dangerous environments where attacks on healthcare personnel and facilities may be a part of the tactical strategy. Expected and traditional protections under International Humanitarian Law and the Geneva Convention are no longer guaranteed [3]. Limiting civilian collateral damage may not be high on the priority list and, in worst cases, inflicting intentional mass casualty events against vulnerable civilian populations may even be a tactical consideration.

CTM emphasizes and highlights the risks of intentional attacks on population health, the risk to responders and medical systems, and incorporates high threat training such as Tactical Emergency Casualty Care as part of the program [4]. Very similar to our history in the changing face of global humanitarian crises, new competencies must be developed for CTM, along with designated multidisciplinary training programs. While traditional combat or battlefield medicine still differs significantly from CTM, when civilians and civilian health infrastructure and systems are intentionally attacked and injured, lessons learned in the sphere of CTM are transplantable and relevant in today’s war.

## Discussion

### Hybrid warfare: what history tells us

The term hybrid warfare was first proposed in 2007 by Frank Hoffmann, a researcher in emerging conflicts in the twenty-first century. He suggested that “we are entering a time when multiple types of warfare will be used simultaneously by flexible sophisticated adversaries” [5]. He forecasted that non-state actors would employ hybrid forms of warfare along with conventional warfare to achieve their goals.

Whereas various definitions of hybrid warfare have emerged, it is usually seen as a combination of conventional warfare, irregular warfare, terrorism and different types of state threatening criminality. These multimodal activities are generally co-ordinated to achieve synergistic effects [5]. The very core of hybrid warfare is a lack of ethical or judicial restraint which creates a direct negative impact on the civilian population.

The history of what led to the development of chemicals as a first-use weapon in war has a unique and troubling history. In both the pre-World War II decades and subsequent successes of the Soviet Union as a global power, more money was spent on developing, producing, and equipping chemical weapons than on all public education, and many more times than on advancement of culture [6]. Syria in the same time period saw themselves in a precarious position compared to the surrounding middle eastern countries, all with large armies and threatening hostilities. Starting in 1970, Syria turned to Russia for both training and supplies and subsequently used their stockpile of mustard gas, sarin and VX in Assad’s long-term war against his own people. Assad used chemical weapons against the Syrian people at least 50 times, assuring the continuation of the Assad regime [7]. Additionally Russian forces under the guise of being a designated “UN sponsored peacekeeping force” widely used chemical weapons against the Syrian civilian population, also ensuring Assad’s reign. Various studies documented that chemical weapon violations in Syria were purposefully directed toward civilians, citing extreme disparity in direct deaths from major chemical weapons with 97.6% of victims being civilians and only 2.4% being combatants, evidence that attacks were indiscriminate or targeted civilians directly [7].

While there are response differences between hybrid warfare and terrorist attacks, including the prolonged strain on the healthcare system from continued hostilities over a long period of time, this intention to harm civilians in war and the healthcare repercussions of such actions has a lot of common ground with more traditional terrorism. It is therefore possible to use the CTM framework to scientifically analyze past events, as well as to use the CTM cycle in the work of Response, Recovery, Mitigation and Preparedness [4].

### Targeting healthcare: modern warfare strategy?

The full-scale Russian invasion and extensive combat operations in Ukraine, which constitutes the largest armed conflict in Europe since World War II, were launched on February 24, 2022. Exactly like Syria, the Russian military claimed their military intervention in Ukraine was being performed by designated UN “peacekeepers” [8]. Since the start of the Ukrainian war, multiple attacks against healthcare workers and facilities have been reported [3]. Ambulances have been shot at, hindered, used as Trojan horses and targeted in secondary attacks, something witnessed in other acts of terrorism [9, 10], and multiple hospitals have sustained indiscriminate or deliberate attacks, all certifying that this invasion constitutes hybrid warfare.

Incidents include the maternity hospital airstrike in Mariupol (March 9, 2022) and the hostage taking of reportedly 500 people in the Regional Intensive Care Hospital in the same city (March 15, 2022). As of November 28, 2022 the World Health Organization (WHO) has verified 715 attacks against healthcare during this war, inflicting 100 deaths and injuring 129 people. These attacks included damage to or destruction of facilities (*n = *630), medical transport (91) and supplies (174), as well as attacks on patients (24) and personnel (61). Eighty-four percent of the attacks on Ukrainian healthcare facilities have been performed with heavy weaponry [11]. Beyond the direct effects of injuries, deaths, and destruction of health infrastructure, there are wider disruptions of routine care, maternal and child health, complex care for those with cancer or needing kidney dialysis, and unchecked spread of infectious diseases, including COVID-19, tuberculosis, and HIV/AIDS [3].

Attacks against hospitals have occurred during multiple armed conflicts, such as the Chechen War (1999–2009), Syria Civil War (2011-present) [12] and Tigray War in Ethiopia (November 2020-present) [13], but it seems that such attacks are deployed systematically and on an unparalleled wide scale in Ukraine. In fact, the WHO recently stated that the targeting of healthcare facilities has become part of the strategy and tactics of modern warfare [14].

Through their Health Care in Danger initiative, the International Committee of the Red Cross (ICRC) aims to address the issue of violence against patients, health workers, facilities and vehicles, and to ensure safe access to and delivery of healthcare in armed conflict and other emergencies. Between 2015 and 2017, the ICRC recorded more than 1,200 incidents of violence against healthcare in the 16 countries where they operated during that time [15]. The most frequent acts against healthcare during this period included destruction of and damage to medical facilities or medical transport, use of explosive weapons, forced interference in a health facility, threats, and denial of access to the sick and wounded. In total, there were 3,290 deaths, 1,750 wounded, 170 abductions, 100 detainments and 10 victims of rape [15]. More than half of the attacks occurred within a healthcare facility, further highlighting the need to target harden such vulnerable soft targets [16, 17].

A quarter occurred in public places, at checkpoints or at border crossings, where both healthcare workers and the sick and wounded are more vulnerable. One important measure that can safeguard timely access to the sick and wounded is therefore to improve acceptance of and respect for pre-hospital and ambulance services on the part of state authorities, non-state armed groups and local communities [15].

### Nuclear and chemical targets

The early days of the Russo-Ukrainian war were not only the stage of attacks against healthcare institutions, but there have also been multiple attacks against schools, humanitarian (evacuation) corridors, churches, civilian shelters, residential buildings and the energy infrastructure.

Attacks on nuclear power facilities run counter to article 56 of Additional Protocol I to the Geneva Conventions, but despite the potential catastrophic risks to human lives, nuclear power plants have been the epicenter of military engagement [18]. These include the battle of Chernobyl and the attack on the Zaporizhzhia Nuclear power plant, where Russian forces repeatedly fired heavy weapons in the direction of the reactor buildings [19].

Nuclear reactors become targets during military conflict and, over the past 3 decades, have been repeatedly attacked during military air strikes, occupations and invasions. They are particularly vulnerable to cyberattacks [20]. If safety systems or spent fuel pools are heavily damaged, this may cause a core meltdown and lead to widespread radioactive contamination.

Chemical plants have also been targets of this war. At the time of this writing, at least two chemical facilities in Ukraine have been attacked. On March 21, 2022, Russian forces shelled a chemical plant near the north-eastern Ukrainian city of Sumy, causing an ammonia leak [21]. Although the leak was fairly small and there were no reported casualties, this event highlights the risk of the attack of industrial targets during this conflict. On April 5, 2022 an industrial-sized hydrochloric acid tank was blown up in Rubizhne in the Luhansk region [22].

Undoubtedly, attacks on both nuclear and chemical targets have the potential to indiscriminately injure and kill large numbers of civilians.

### Resurfaced use of chemical weapons

Another concern is the resurfaced use of chemical weapons and other particularly damaging weapons such as cluster munitions, which are both banned under the Chemical Weapons Convention and the Convention on Cluster Munitions [23].

On April 13, it was reported that chemical weapons have been used by Russian forces attacking the Ukrainian port of Mariupol. A “poisonous substance of unknown origin" was allegedly released by a drone [24]. This report has not been independently verified.

Cluster munition has been used on multiple occasions to date, of which some impacted healthcare facilities [23]. The use of drones, chemical weapons, and the civilian healthcare response preparedness and toxidrome recognitions are core research and discussion topics within counter-terrorism medicine [25–30].

### The Ukrainian pivot

The current humanitarian disaster in Ukraine has been evolving since the annexation of Crimea in 2014 and is a devastating example of how healthcare and civilians are affected, from an initial grey zone situation to a complete hybrid war. Collecting and verifying data during wars, conflicts and terrorist attacks have historically been a difficult task but modern technologies and methodologies and improved global connectivity allows for better documentation of events [3]. The Ukrainian Healthcare Center (UHC) is a think tank which played a significant role in the health system reform in Ukraine in 2016–2019. They participated in the reform design, adoption of the legislation, establishment of the National Health Service of Ukraine, development of the eHealth system, the organizational transformation of the Ministry of Health, health reform communication, and implementation of the reform at a local level [31].

After the 2022 Russian invasion of Ukraine, the UHC team concentrated on war-related initiatives, collecting, verifying and documenting data points similar to those published in many recent CTM research articles: attack modalities and weapons used, death and injury tolls, and damage sustained to critical health infrastructure [9, 10, 17].

Demand in Ukraine for tactical emergency care training, a core staple of Counter Terrorism Medicine training, also surged since the outbreak of war, further drawing parallels and similarities between CTM and the current war [32, 33].

## Conclusion: not terrorism, not war

Bridging events that involve organized attacks against healthcare from either hybrid warfare or terrorism, CTM remains a rapidly emerging field of Disaster Medicine. While most civilian healthcare systems and personnel would not willingly choose to actively seek out environments of threat, threat may inadvertently seek them out and with potentially devastating consequences. CTM aims to better prepare medical systems in mitigating the risks of intentional attacks, and in a world where domestic terrorism is on the rise and where war is at our doorstep, CTM appears to be more relevant than ever today.

## Data Availability

Data sharing is not applicable to this article as no new data were created or analyzed in this study.

## Abbreviations

CTM: Counter-terrorism medicine
UHC: Ukrainian Healthcare Center
